# Causes of Missed Nursing Care During COVID-19 Pandemic: A Qualitative Study in Iran

**DOI:** 10.3389/fpubh.2022.758156

**Published:** 2022-04-13

**Authors:** Ali Safdari, Maryam Rassouli, Raana Jafarizadeh, Fatemeh Khademi, Salman Barasteh

**Affiliations:** ^1^Student Research Committee, Baqiyatallah University of Medical Sciences, Tehran, Iran; ^2^Cancer Research Center, Shahid Beheshti University of Medical Sciences, Tehran, Iran; ^3^Department of Medicine, Ardabil Branch, Islamic Azad University, Ardabil, Iran; ^4^Department of Nursing, Faculty of Nursing, Arak University of Medical Sciences, Arak, Iran; ^5^Health Management Research Center, Nursing Faculty, Baqiyatallah University of Medical Sciences, Tehran, Iran

**Keywords:** missed nursing care, qualitative study, COVID-19, quality of care, nursing care, pandemic

## Abstract

**Background:**

The unpredictable and variable nature of COVID-19 and the lack of healthcare resources has led to inadequate care for patients. This study aimed to explain the causes of missed nursing care during the COVID-19 pandemic from the perspective of Iranian nurses.

**Method:**

This qualitative study was conducted using semi-structured interviews with 14 nurses caring for patients with COVID-19 in three hospitals in Iran. Sampling was performed by the purposive method. Data were analyzed using the conventional content analysis method. The interviews were first recorded and transcribed, and then the data were analyzed using the Elo and Kyngas method. Data management was done with MAXQDA software version 10. To achieve trustworthiness, the criteria presented by Lincoln and Guba were used.

**Findings:**

A total of 14 nurses participated in the study. The mean age of participants was 31.85 ± 4.95 years, and the mean number of years of work experience was 7.71 ± 4.44. Eleven participants were women. Among all participants, nine had a bachelor's degree and five had a master's degree. Four nurses had fixed shifts, while ten nurses had rotating shifts. The causes of missed nursing care were categorized into 4 groups. The category “unfulfilled care” comprised the reasons for forgetting care, neglecting care, arbitrary elimination of care, and compulsory elimination of care. The category of “care at improper time” consisted of interference of the care in patients' daily activities and interference with other healthcare providers' activities. The “incomplete cares” category comprised failure to complete the care period in hospital, interruption in care, and discontinuance of care after patient discharge. The last category, “incorrect care,” consisted of providing care regardless of the nursing process, providing care by unqualified professionals, and providing trial-and-error care.

**Conclusion:**

This study illustrates an understanding of the causes of missed nursing care during the COVID-19 pandemic from the perspective of nurses. The increasing demand for care caused by the pandemic and problems in the work environment has led to the failure of nurses to provide complete, correct care and sometimes miss parts of care to patients. Therefore, nursing policymakers and managers should develop and implement appropriate care protocols and instructions to minimize missed nursing care.

## Introduction

The COVID-19 pandemic has been described as the largest global challenge since World War II (1). More than any other pandemic in recent years, it has led to societal turmoil and death around the world. Iran also has had one of the highest rates of infection and mortality since the beginning of the COVID-19 pandemic, and the vulnerability of healthcare providers has been a serious challenge to the country's health system (2). The pandemic situation has posed many challenges for healthcare providers, especially nurses (3).

The pandemic has exacerbated the long-term challenges faced by nurses concerning inequalities (e.g., low payment and a high number of employed women), improper work conditions, and excessive work workload. A clear example is an increase in vacant capacity, absenteeism, displacement, and intention to leave reported before the pandemic, with staff having reported feeling “broken,” “exhausted,” and “on one's knees” (4). Beyond issues related to workload and stress, the serious impact of the pandemic can be seen with the cost to life in many nurses working during COVID-19 having lost their lives (5).

In particular, nurses in Iran faced several problems during COVID-19, such as heavy workload (6), psychological distress [e.g., low resilience (7) and high level of job stress, fear, and anxiety (8, 9)], “turmoil,” and “lack of support and equipment (9).” All of these and the increasing volume of patients and the complexity of patient care make it impossible for nurses to provide all the care necessary to patients. In these situations, nurses may ignore, delay, or even eliminate some parts of care completely (10).

Missed nursing care (MNC) was first introduced in a study (2006) that identified care (such as physical and emotional care) that was unfinished, delayed, or not performed (11). The introduced model illustrated the various attribute categories that contribute to missed nursing care; antecedents within the care environment that facilitate or inhibit the practice of nursing; elements of the nursing process; internal perceptions and decision processes; care that is provided as planned; care that is delayed or omitted; and patient outcomes. Environmental factors include labor resources available to provide patient care, material resources accessible to assist in patient care activities, and various relationship and communication factors that have an impact on nurses' ability to provide care (12).

The recent pandemic situation caused nurses not to be able to provide patient- and family-centered care due to changes in patient management, differences in care, and restrictions meant to prevent the spread of the disease (13). Given the nature of the infection, nurses were compelled to prioritize nursing care tasks that addressed patients' oxygenation status, positioning to maximize lung expansion, and the administration of antibiotics and antiviral drugs over other nursing care tasks such as the maintenance of personal hygiene, nursing surveillance, and other communications and interactions with patients (14).

In this critical period, however, nurses also faced challenges such as safety concerns, the threat of infection, infection transmission to their family members, moral distress, increased workload, and short training times. All these factors had a negative impact on nurses' health (15). The COVID-19 pandemic has exacerbated the imbalance between limited nursing resources and increased patient needs (16).

Experience with past pandemics has shown that the initial planning for patient care is not feasible due to the unpredictability of environmental conditions (17). Despite severe job pressure and changes in nurses' work environment, the occurrence of MNC is not unexpected (10). Therefore, due to changes in nurses' work environment and differences in the frequency, type, and causes of MNC in different countries during the COVID-19 pandemic, further studies in developing countries seem necessary. Understanding the factors and causes that contributed to MNC during the pandemic is essential to formulating relevant organizational measures to prevent the recurrence of MNC that may potentially affect the health and wellbeing of patients (10). A previous study conducted in Iran showed the presence of MNC in the health system of Iran to be undeniable (18). Accordingly, this qualitative study was conducted to explore the causes of MNC during the COVID-19 pandemic from the perspective of nurses in Iran.

## Method

### Design and Setting

This qualitative content analysis study explored the causes of MNC during the COVID-19 pandemic from the perspective of Iranian nurses. In-depth and semi-structured interviews with participants were used to collect data. Data were collected from December 2020 to February 2021 in three hospitals in Iran: Baqiyatallah Al-Azam in Tehran and Amir Al-Momenin and Ayatollah Khansari in Arak. The content analysis aims to organize and extract meaning from the compiled data and to draw significant conclusions from it. The use of qualitative methods provides the possibility of discovering confidential information that can only be revealed through this way (19). This approach makes it possible to describe the perception and insight of healthcare givers concerning a less-perceived event, such as the COVID-19 pandemic (17). The study report presented using the consolidated criteria for qualitative reporting research (COREQ) (20) (Supplementary File 1).

### Participants and Sampling

A total of 14 nurses were selected in the study using purposive sampling with maximum variation (Table 1). No participants were excluded from the study. To achieve a comprehensive understanding of the phenomenon, the participants' triangulation method was used (21). In this way, nurses who had sufficient experience in caring for COVID-19 patients were selected from different wards in three different hospitals. Nurses with at least 1 year of experience as a clinical nurse, at least 2 weeks' experience in providing care for COVID-19 patients, and willingness to participate in the study were included in the study. The exclusion criterion was an unwillingness to continue participating in the study. No participants refused to participate or withdrew after giving their consent. The first participant was a 35-year-old female nurse who had experience caring for COVID-19 patients in the ICU. The first interview was transcribed by the AS and listened to several times by the research team. The interview was critiqued. AS re-interviewed this participant. Interviews were conducted by appointment at a convenient place in the nurses' workplace. Interviews were conducted by AS, a 32-year-old male nursing graduate student with 5 years of nursing experience. Before the interview, he had participated in a qualitative study course and had received training in the qualitative study, interviewing, coding, and reporting by SB. The MR and SB have enough experience in performing and writing qualitative studies. The interviews were recorded with an mp4 recorder.

**Table 1:** Demographic information of participants in the research.

**Pseudonym assigned**	**Occupational [Ward/ shift/ Experience (years)]**	**Life situation**
Anita	General/ rotating/ 6	30 years old, Single, female, bachelor, Living with her spouse, childless
Farnaz	General/ Morning & evening/ 5	28 years old, Single, female, bachelor, Living with mother. The father died a year ago, poor economic situation and low family expenses
Akram	ICU/ rotating/ 10	35 years old, female, Master degree, Living with her spouse, having one child, average economic status
Sanaz	ICU/ rotating/ 4	29 years old, Single, female, Master degree, Living alone, recently migrated to Tehran from Khorramabad, socializing a lot with friends
Hojjat	Emergency/ rotating/ 5	29 years old, Single, male, Master degree, Living alone, doing sports and entertainment, selling mobile phones as a second job
Faezeh	General/ night/ 8	33 years old, Married, female, Master degree, She lives with her husband, is childless, and her husband is a nurse in another hospital
Reza	Emergency/ rotating/ 15	39 years old, Married, male, Master degree, Living with his wife, having two children, having sense of humor, average economic status
Ziba	General/ rotating/ 7	32 years old, married, female, bachelor, Living with her husband, childless, originally Gilaki
Shila	General/ rotating/ 7	28 years old, Single, female, bachelor, Living with parents, having a military father, rigidly disciplined
Mahsa	General/ morning/ 10	33 years old, Married, female, bachelor, Living with her husband, serializable, having sense of humor
Zohre	ICU/ rotating/ 18	44 years old, Divorced, female, bachelor, Living with a 12 years-old daughter, divorced for 10 years, frowns a lot, loves reading books
Azin	General/ rotating/ 8	33 years old, Single, female, bachelor, Living with parents, prodigal, has recently broken a relationship with her boyfriend
Aida	General/ rotating/ 3	27 years old, Single, female, bachelor, Living alone, average economic status
Hamid	Emergency/ rotating/ 2	26 years old, Single, male, bachelor, Living with two other friends in a bachelor pad, poor economic status

### Data Collection and Procedures

Data were collected through individual, face-to-face, in-depth semi-structured interviews by the first researcher. Field notes were taken during the interviews. Interviews were conducted in a quiet place preferred by the interviewees, which was mainly the care unit where the interviewee worked. The researcher introduced himself and the objectives of the research obtained permission to record conversations and the possibility of re-referral to validate the data and then asked the participants to describe their experiences. The main research question was “What were the reasons for the missing of nursing care during the COVID-19 pandemic?”. Also, exploratory questions based on participants' experiences were asked to obtain more detail about the causes of MNC. Examples of these questions were “What care is missed while caring for COVID-19 patients?”, “How much of your care was missed during this pandemic?”, “What factors contributed to the missing of nursing care during the COVID-19 pandemic?”, and “How and under what circumstances was this care missed?”. The average interview time was 52 ± 5.21 min and was continued until data saturation was met. The interview guide is reported in Supplementary File 2. Data saturation occurs when there is enough information to repeat the study, when the ability to obtain additional new information is obtained, and when most coding is no longer possible (22).

### Data Analysis

Data we analyzed using the conventional content analysis method based on the method proposed by Elo and Kyngas in preparation, organization, and reporting stages (Table 2) (22). The interviews were transcribed and read several times to gain an understanding of the entire interview. Initially, we read the content of interviews several times to obtain a general understanding. Following that, we identified the meaning units and the primary codes. Afterward, similar primary codes were condensed and merged into subcategories, and the main categories were extracted [(23) Table 2]. For encoding and data management, MAXQDA software version 10 was used (24). The creation of codes and categories in MAXQDA is controlled by the user, who can create codes before, during, or after the material is analyzed. This software can use different meaning units in the coding process to associate variables to the texts; therefore, codes can be associated with the selected text. During or after coding, the user can check the correspondence between a set of codes and texts. However, this software does not greatly differ from similar software. Researchers mainly perform the process of analyzing and coding the qualitative data based on familiarity with one of the qualitative data analysis software (25). Finally, categories and subcategories were identified from the data. An example of data analysis is provided in Table 3. During the coding process, a code book was created and the first author used the code book to encode the rest of the interviews.

**Table 2:** Summary of Elo and Kyngas qualitative method.

Preparation	Analysis unit selection	Semantic units were identified by transcribing the interviews to text and explicating the content.
	Finding the logical link between the data and the subject	Reading the text of the interview several times to gain a comprehensive understanding and prolong engagement with the data.
Organizing	Creating an analytical framework	Forgetting care, neglected care, arbitrary elimination of care, compulsory elimination of care, delay in care, interference of care with patients' daily activities, interference with other healthcare providers' activities, failure to complete the care period in the hospital, interruption in care, discontinuing care after patient discharge, providing cares regardless of the nursing process, providing care by unqualified professionals, and providing trial-and-error care were included as the main subcategories in the matrix.
	Extracting data from the content	The main classes were formed based on conceptual and logical relationships with other classes.
	Grouping	Similar codes were merged based on differences and similarities.
	Classification	The formed groups were classified based on their differences and similarities.
	Abstracting	Similar categories were placed in the main categories of the matrix.
Reporting	Illustrating data collection, data analysis, and each of the main categories in the form of final report, an article in a journal, etc.	

**Table 3:** An example of data analysis.

**Categories**	**Subcategories**	**Primary codes**	**Quotation**
Unfulfilled care	Forgetting care	Forgetting to communicate with patients	I have to communicate more with patients before COVID-19. Now I like to talk to them. But we are so busy that sometimes I forget to talk to patients.

### Trustworthiness

Guba and Lincoln's criteria of credibility, transferability, dependability, and confirmability were considered to achieve trustworthiness (26). For credibility, maximum diversity in sampling, relationships with the participants, continuous observation, and reflections on the credibility of the researchers (all nurses participated in caring for patients with COVID-19 during the pandemic) were used. Enough time was spent collecting and immersing the data. The interviews text and codes were sent to participants and their feedbacks were obtained as member check. And AS and SB checked and re-checked the codes to achieve peer check. Then, to achieve dependability and confirmability, all of the authors tried to achieve a definitive organization of data together. No major disagreements were noted but vague phrases and sentences were discussed and agreed upon. Also, any differences between codes, subcategories, and categories were discussed until a consensus was reached. Due to the ease of data grouping, the research team members mutually agreed that the data were well-saturated. For transferability, a rich analytical description of the context, methodology, and limitations and also maximum variation sampling were presented.

### Ethical Considerations

This study was approved by the ethics committee of the Baqiyatallah University of Medical Sciences, Tehran, Iran, with the code: IR.BMSU.REC.1399.518. Written and oral consent as a part of the Helsinki Declaration was also obtained from all participants for the recording of their interviews. Participants voluntarily joined the study and were ensured of the confidentiality of their information and the right to withdraw from the study.

## Findings

A total of 14 nurses working in COVID-19 centers participated in the study. Table 1 shows the demographic information of the participants. The mean age of participants was 31.85 ± 4.95 years, and the mean number of years of experience was 7.71 ± 4.44. The number of female participants was 11 among all participants, nine had a bachelor's degree, and five had a master's degree. All but 10 nurses had fixed shifts; those 10 had rotating shifts. Data analysis led to the emergence of four main categories: “unfulfilled care,” “care at improper times,” “incomplete care,” and “incorrect care” (Table 4).

**Table 4:** Categories and subcategories of missed nursing care types in COVID-19.

**Category**	**Subcategory**
Unfulfilled care	Forgetting care
	Neglected care
	Arbitrary elimination of care
	Compulsory elimination of care
Care at improper times	Delay in care
	Interference of care with patients' daily activities
	Interference with other healthcare providers' activities
Incomplete care	Failure to complete the care period in the hospital
	Interruption in care
	Discontinuance of care after patient discharge
Incorrect care	Providing care regardless of the nursing process
	Providing care by unqualified professionals
	Providing trial-and-error care

### Category 1: Unfulfilled Care

Unfulfilled care refers to the essential care that should have been done but was left undone for any reason. This category includes “forgetting care,” “neglecting care,” “arbitrary elimination of care,” and “compulsory elimination of care.”

#### Subcategory 1.1: Forgetting Care

Some nurses cited that they fail to remember some parts of care. In other words, they were unable to think of or recall that care. Reasons for forgetfulness included fatigue, lack of adequate care experience, extensive patient care needs, and a variety of symptoms. Most nurses cited forgetting as one of the reasons for unfulfilled care due to poor awareness or a high volume of activities. Farnaz, a 28-year-old female nurse who worked in the general ward, said that “*Our ward was previously a cardiac unit. I used to give patients their pills, and then my work was done. Now, however, all my patients have a reserve bag mask or use noninvasive ventilation devices. I really inadvertently forget some parts of care.”*

#### Subcategory 1.2: Neglecting Care

Despite their knowledge and awareness of some parts of care, nurses sometimes refuse to perform them for fear of being infected with the virus. Anita, a 30-year-old female nurse with 6 years of experience in the general ward, said that “*Even though some of our colleagues knew that the patient's secretions had increased, they did not do the suction until the patient's O*_2_ *saturation dropped or it became difficult for the patient to breathe, because they were afraid of being infected by the patient's secretions.”*

#### Subcategory 1.3: Arbitrary Elimination of Care

Some of the MNCs reported by nurses followed disagreements and different care perspectives and poor teamwork between physicians and nurses. Reza, a 39-year-old male nurse with a master's degree who worked in the emergency ward, said that “*[…] It is not necessary to control the blood pressure of a young patient who has no problem every 2 h […] patients are also bothered […]. that's why we just charted it in the sheet without doing that […].”*

#### Subcategory 1.4: Compulsory Elimination of Care

Lack of equipment and human resources and inadequate hospital infrastructure caused the failure of nurses to provide some nursing care to hospitalized patients. Mahsa, a 33-year-old nurse with a bachelor's degree, has experienced that some mandatory care has been missed “*We had shifts where the oxygen consumption of the hospital was so high that we had to cut off the oxygen of some patients so that we could use it for anesthetized and intubated patients […].”*

### Category 2: Care at Improper Times

This category includes three subcategories: “delay in care,” “interference of care with patients' daily activities,” and “interference with other healthcare providers' activities.”

#### Subcategory 2.1: Delay in Care

Prioritizing nursing care for critically ill patients and the high workload of nurses delayed the provision of timely care to other patients. Anita, a 30-year-old female nurse with 6 years of experience in the general ward, said that “*Most patients complained of pain. Maybe at the moment that they were asking us for help, we could not take care of them at that time, because first, we had to pay attention to the needs of the critically ill patients.”*

#### Subcategory 2.2: Interference of Care With Patients' Daily Activities

Interference between nursing care times and patients' daily routines such as sleeping and eating was another cause of MNC. Sanaz is a 29-year-old female nurse with a master's degree in rotating shift said “*One issue that patients complained about was that nursing care did not have specific timing, and nurses woke them up to do different things, which led to disrupted peace and rest.”*

#### Subcategory 2.3: Interference With Other Healthcare Providers' Activities

Interference of care with the activities of other healthcare team members due to time constraints led to failure to provide some parts of nursing care. Hamid, a 26-year-old male nurse with a bachelor's degree, said “*When doctors enter the ward, they are in a hurry to visit and leave the ward […]. we have to perform and finish the care quickly or leave it unfinished until the doctors complete their visit!”*.

### Category 3: Incomplete Care

It includes three subcategories: “failure to complete the care period in the hospital,” “interruption of care,” and “discontinuance of care after discharge.”

#### Subcategory 3.1: Failure to Complete the Care Period in the Hospital

Discharging patients who had only partial improvement to admit new critically ill patients led to MNC. Zohreh, a 44-year-old nurse with a bachelor's degree, said “*[…] In order to be able to admit new critically ill patients from the emergency ward, we had to discharge patients who had only partially improved their general condition according to the doctor (opinion) before completing the treatment so that a bed would be empty for a new patient […].”*

#### Subcategory 3.2: Interruption of Care

Weakness in observing continuous and regular nursing care to achieve the desired results led to the provision of intermittent care to patients. Faezeh, a 33-year-old female night work nurse with 8 years of experience in the general ward, said “*[…] The chest physiotherapy that we do for intubated and anesthetized patients is not done at regular periods. Certainly if it were done regularly, its effect would be multiplied” (P6)*.

#### Subcategory 3.3: Discontinuance of Care After Discharge

The presence of some long-term complications caused by COVID-19, even months after patients become infected, necessitates follow-up after treatment and post-discharge care. Mahsa, a 33-year-old nurse with a bachelor's degree, has experienced that discontinuance of care after discharge leads to missed nursing care “*You see, the care we provide is limited to the duration of time that patients are hospitalized. Then they are discharged only with a series of general training, and it is thought that they are enough.”*

### Category 4: Incorrect Care

This category includes “providing care regardless of the nursing process,” “providing care by unqualified professionals,” and “trial-and-error in providing care.”

#### Subcategory 4.1: Providing Care Regardless of the Nursing Process

Providing care without using the nursing process as a problem-solving approach with a logical result led to missed principled and rational care provided to the patients. Zohreh, a 44-year-old nurse with a bachelor's degree, illustrates that providing care regardless of the nursing process leads to a missed of care “*[…] Some days we have so much work that we just have time to do routine tasks, like giving patients' medicine and suctioning. There is no opportunity to provide for other patients' needs or doing our care based on a specific nursing diagnosis.”*

#### Subcategory 4.2: Providing Care by Unqualified Professionals

Improper delegation of nursing duties and ambiguity in the roles and responsibilities of different levels of nurses such as the employment of retirees and nursing students led to MNC in patients with COVID-19. Akram, a 35-year-old female nurse, illustrates how unqualified professionals lead to missed nursing care “*[…] I told one of the nurses' assistants to perform a gavage for one of the patients. She did not notice, and she fed him a full glass of carrot juice; thereafter, when the NIV mask was put on him, the patient aspirated […].”*

#### Subcategory 4.3: Providing Trial-And-Error Care

The lack of a standard and approved protocol due to the emerging and unknown conditions of COVID-19 led to a constant change in medication and care administered to patients through trial and error. Shahla, a 33-year-old female nurse caring for patients with COVID-19, experienced that “*[…] We used to think that it would be better to put all of our patients in a semi-sitting position so they could breathe better, but now our doctors also order lateral and even prone positions” (P12)*.

## Discussion

In this study, the causes of MNC for COVID-19 patients were explored through the experiences of nurses. This study offers new insight into healthcare decision-making for these patients. One aspect of quality nursing care is the amount of care that is omitted. In 2009, Beatrice J. Kalisch et al. showed that MNCs and the reasons for them (labor resources, material resources, and communication) were common. Job title (e.g., registered nurse vs. nurse assistant), shift work, absenteeism, perceived staffing adequacy, and workload were significantly related to missing the care (27). Other studies have shown that staff adequacy and a supportive work environment for nurses are related to a lower prevalence of MNC (28, 29). Nurses who work in favorable working environments have reported a lower prevalence of MNC.

Participants in this study reported that MNCs occurred in the care of COVID-19 patients because of unfulfilled care. It seems that factors caused by changes in the work environment, namely, the formation of new care teams, lack of resources, and heavy workload, lead to nurses forgetting some of the essential care of COVID-19 patients; ultimately, the care will be missed. For example, in this study, patients' emotional and psychological support was forgotten, which is in line with the findings of other studies (30, 31). Therefore, it is necessary to review nurses' work schedules and shifts in a scientific and reasonable way to optimize their performance and focus on the lack of care during the pandemic.

Another part of unfulfilled care was neglected care. Neglected care was also considered as unfulfilled care by Palese et al. and Kalisch (11, 30) but the main neglected care during the COVID-19 pandemic was psychological and spiritual care (32, 33). Dutra et al. argued that working in environments with structured work processes and a sufficient number of specialists can reduce neglected care and help with care planning, patient education, and emotional support, which are not always valued in the care process (34). Nurses often overlooked this type of care due to the possibility of contamination with COVID-19 or work-related fatigue.

Part of patient care was arbitrarily eliminated because of different care perspectives, teamwork, and poor supervision. Since the atmosphere of the healthcare system in Iran is traditional and unscientific, the relationship between physician and nurse is hierarchically vertical. The opinions of nurses are not approved or accepted, even concerning nursing care (33). It seems that a change in the inefficient hierarchical culture and alignment between the perceptions, attitudes, and performance of different healthcare team members would lead to improved interdisciplinary communication and collaboration.

According to the participants, the lack of resources and insufficient hospital infrastructure inevitably led to unfulfilled parts of care. Most studies by Kalisch et al. cited hospital resources as one of the causes of MNC (11, 35). A previous study conducted in hospitals in Iran also emphasized the lack of facilities and old and defective equipment as factors affecting MNC (36). However, “staff and resource adequacy” was not found as a significant predictor of MNC during the COVID-19 pandemic (37). Due to the weakness in public vaccination and the collapse of hospitals in Iran at the time of the study, the existence of such findings is not unexpected.

Other MNCs reported by nurses included care that was not performed on time or interfered with the activities of patients or other caregivers. Similarly, Kalisch made it clear in 2006 that delay in care is one of the most influential factors in MNC (11). Also, another study conducted in Iran in 2018 mentioned that delays in accessing prescribed medications during one shift lead to prescribing medications in the next shift (18). Abdelhadi et al. also admitted that nurses delayed care they thought was urgent (38).

Interference of care with patients' daily activities occurs mainly due to the lack of effective communication between the nurse and the patient. As reported in Kalisch's studies, weaknesses in effective communication with medical staff are often a dimension of MNC (16). When nurses could not communicate and focused exclusively on tasks such as medication, patients remained unrelated to basic care needs (39). In studies by Marsh et al., nurses reported most missed care in the communication and preparation categories (40). For example, ignoring patients' comfort and relaxation following the interference of nursing care with patients' bedtime or eating was part of the missed care, which is in line with the study of Jangland et al. (41). It seems that environmental factors such as noisy equipment (device alarms and oxygen manometer), uncoordinated care interventions, and the noise of personnel and other patients affect patients' activities.

Nurses cited other MNCs that included “incomplete care” in the hospital and early discharge of patients due to the limited number of hospital beds and the need to provide services to new critically ill patients. The COVID-19 pandemic has affected the process of patient care and treatment, including triage, admission, discharge, and the extent of services provided to patients (42). However, efforts to limit the duration of hospital stay and the tendency to provide outpatient care have moderated the accuracy of care for COVID-19 patients (37). Nurses expressed some causes, namely, the fear of virus infection and the disproportion between nurses and patients as factors affecting interrupted care.

Comprehensive care is a key goal in providing nursing care. The opposite point is “interruption in care”. Lasater et al. noted that most nurses report that their work is frequently interrupted or delayed by inadequate staff, and one-third of nurses report interruptions or delays due to lack of supplies, namely, medications and broken equipment (43). According to a review by Monteiro et al., 88.9–90% of interruptions lead to negative consequences such as delayed treatment and loss of focus (44). On the other hand, Hall et al. have shown that interruptions do not always lead to side effects, and some may have a positive effect on the performance of a specialist and caregiver, because some interruptions may increase the safety, comfort, and accuracy of nurses' duties (45).

Another cause of MNC was the “discontinuance of care by patients after discharge” during the pandemic. Kalisch et al. also mentioned “planning for patient discharge” as one type of MNC (46). Conversely, in the study of Lee et al., patient discharge planning and teaching were less missed (47). However, the importance of post-discharge and follow-up care in patients with COVID-19 seems to be inevitable due to long-term complications, even months after recovery.

Failure to perform nursing care based on the nursing process is another cause of MNC. Kalisch et al. mentioned that the element of the nursing process is part of MNC (12). The results of Tosun et al. showed that some nurses using the nursing process during the COVID-19 process had difficulties with it and often had difficulty in implementing care programs (48). In Labrague's study, interventions that required planning were more often missed (41). It seems that educating nurses, changing their attitude toward patient care, clinical judgments, and allocating more time to care can prevent the occurrence of MNC.

Providing care by unqualified professionals threatens care standards. The inadequate delegation was one of the most important problems in unqualified professionals providing care during this pandemic. Inadequate delegation of nursing duties can lead to negative (real or potential) consequences for patients. Which may lead to MNC such as neglect of rotation, bathing, timely feeding of patients, skincare, and other nursing care (49). Also, Lange has argued that due to the shortage of health professionals in most low- and middle-income countries, sharing of tasks (change of duties) by non-specialists can play an important role in improving access to mental health services. Sharing tasks involve people who do not specialize in providing healthcare. According to Lange, the use of non-specialist health workers with shorter training increases the provision of required care without the need to increase resources and strengthens sustainable health systems (50). Also, the codes of professional ethics are silent on the subject of the duty of care in the event of an outbreak of infectious disease, so there is no guidance on what to expect from healthcare providers or how to deal with the duty of care in the face of danger (51). Therefore, it seems that the utilization of these unqualified professionals nurses and lack of attention to their basic training has led to missed nursing care and endangered the safety of patients.

“Providing trial-and-error care” was the last subcategory of incorrect care. This is in line with the results of Dehghan-Nayeri et al. (36) in which nurses considered the lack of a standard and approved protocol following the outbreak of an unknown and emergent disease as a factor in providing care by trial and error. Previous care methods had to be constantly replaced with new protocols (52). The result of the Aliyu et al. study also shows that they have been forced to use a trial-and-error approach to prepare staff, and clinical staff have relied on each other and resorted to new workarounds (53). In another study conducted by Dehghan Nairi et al. in 2018 in Iran, trial and error in the learning of nursing students was considered as a factor in the missing of nursing care (35). It seems that many new laws and guidelines for infection control have led to nurses overworking in these conditions and MNC.

## Conclusion

This study illustrates a comprehensive understanding of the causes of MNC during the COVID-19 pandemic from the perspective of nurses. The increasing demand for care caused by the pandemic and the problems of the work environment has led to the failure of nurses to provide complete, correct care, and sometimes to miss parts of care to patients. The results of this study may be generalized to other nursing care centers in the healthcare system of Iran and similar countries. Therefore, nursing policymakers and managers should develop and implement appropriate care protocols and instructions to minimize MNCs.

## Limitations

Due to the qualitative nature of the study, the difficulty in recruiting an adequate number of participants that would allow data saturation was one of the limitations of this study. The presence of the interviewer in the hospital setting was very difficult due to restrictions imposed after the outbreak of COVID-19. Moreover, due to the current pandemic, it was impossible to observe the care process and conduct focus groups to generate data.

## Data Availability Statement

The raw data supporting the conclusions of this article will be made available by the authors, without undue reservation.

## Ethics Statement

Written informed consent was obtained from the individual(s) for the publication of any potentially identifiable images or data included in this article.

## Author Contributions

AS, MR, and SB: study design. FKH: data collection. AS and RJZ: data analysis. AS and SB: study supervision. AS, MR, SB, and RJZ: manuscript writing. SB, MR, AS, and FKH: critical revisions for important intellectual content. All authors read and approved the final manuscript.

## Conflict of Interest

The authors declare that the research was conducted in the absence of any commercial or financial relationships that could be construed as a potential conflict of interest.

## Publisher's Note

All claims expressed in this article are solely those of the authors and do not necessarily represent those of their affiliated organizations, or those of the publisher, the editors and the reviewers. Any product that may be evaluated in this article, or claim that may be made by its manufacturer, is not guaranteed or endorsed by the publisher.
