# Supplementary material for: Causes of Missed Nursing Care During COVID-19 Pandemic: A Qualitative Study in Iran
Source: Front Public Health. 2022 Apr 13;10:758156. doi: 10.3389/fpubh.2022.758156 (PMC9043243; doi:10.3389/fpubh.2022.758156)
Supplement: Supplementary file 2 [file Data_Sheet_2.docx]

**Interview schedule**

**1. Orientation**

The aim of our study was to explain the reasons for the missing of nursing care during the COVID-19 pandemic. To do this, your consent to participate in the study is required, although you can interrupt the interview and withdraw from the study at any time. I have to record the interview. Then I transcribe them. Your name will not be mentioned in the report or article. First, fill out the informed consent form with demographic information including age, sex, years of experience, type of shift, life status, educational status, and marital status.

**2. Primary questions**

Mention "your experience of caring for COVID-19 patients ".

The next questions are based on the participants' experiences. Examples:

What were the conditions of COVID-19 patients?

How many covid-19 patients did you care for daily?

What stress did you experience while caring for these patients?

What was the intensity of the shifts?

How was your experience with the high number of deaths of COVID-19 patients?

**3. Main question**

What were the reasons for the missing of nursing care during the COVID-19 pandemic?

**4. Probing question**

Based on the participants' experiences probing questions were asked. Examples:

What care is missed while caring for COVID-19 patients?

How much of your care was missed during this pandemic?

What factors contributed to the missing of nursing care during the COVID-19 pandemic?

How and under what circumstances was this care missed?

**5. Terminal phase**

The researcher's questions and discussions are over, please let me know if you have any other points or questions.

**Thank you for your participation in this study.**
